# Effect of Gold Nanoparticles on Luminescence Enhancement in Antibodies for TORCH Detection

**DOI:** 10.3390/molecules29235722

**Published:** 2024-12-04

**Authors:** Cuimei Chen, Ping Ding

**Affiliations:** 1 School of Public Health, Xiangnan University, Chenzhou 423000, China; cmc_xnxy66@163.com; 2 Xiangya School of Public Health, Central South University, Changsha 410078, China

**Keywords:** Au NPs, luminol–H_2_O_2_, TORCH, detection

## Abstract

Purposes: To explore the optimization method and application of Au-NP-enhanced luminol––H_2_O_2_ luminescence system in TORCH (TOX, RV, CMV, HSVI, and HSVII) detection. Method: 4.5 × 10^−5^ mmol/L gold nano solution was prepared with chloroauric acid as the reducing agent and trisodium citrate as the stabilizer. After curing for 3 days, Au NPs participate in the luminal–H_2_O_2_ luminescence system to detect TORCH antibodies and establish the cut off value. SPSS 18.0 software was used to analyze the TORCH antibodies detected by the nano-gold-enhanced luminol luminescence method and TORCH kit. Additionally, its detection performance is studied. Results: The results of a paired *t*-test for the absorbance values of samples with and without gold nanoparticles showed that there were statistically significant differences (*p* < 0.001) between the two methods in the detection of TOX, RV, CMV, HSVI, and HSVII. The luminescence values with the addition of gold nanoparticles were significantly higher than those without gold nanoparticles. Using the Au NP–luminol–H_2_O_2_ chemiluminescence method, 127 serum samples were tested for TORCH antibodies. The sensitivities were 84.6%, 83.3%, 90.9%, 85.7%, and 84.6%, while the specificities were 94.7%, 96.5%, 96.6%, 97.3%, and 95.6%, respectively. The sensitivity and specificity of the chemiluminescence method enhanced by gold nanoparticles are significantly improved compared to the chemiluminescence method without enhancers. Conclusions: Au NPs participate in the luminal–H_2_O_2_ luminescent system. The absorbance, sensitivity, and specificity of TORCH antibodies show that Au NPs can enhance the luminol–H_2_O_2_ luminescent system. Au NP–luminol–H_2_O_2_ luminescence system has broad application prospects in the detection of eugenics.

## 1. Introduction

TORCH refers to a pathogen that can cause congenital intrauterine infection and lead to perinatal abnormalities, where T refers to Toxoplasma (tox), O refers to other pathogenic organisms, R refers to rubella virus (RV), C refers to cytomegalovirus (CMV), and H refers to herpes simplex virus (HSV) [1,2,3]. TORCH has as an important factor mother-to-child infection, which has a serious impact on mother-to-child health [4,5]. In order to improve the quality of the birth population, strengthening the detection of TORCH IgM for women before and during pregnancy has potential social and economic benefits [6], which can not only prevent adverse outcomes for mothers and infants, but also realize high-quality interventions for pregnant women and fetuses [6].

Traditional TORCH detection methods mainly rely on etiology, molecular biology, and immunology. However, these methods are limited in laboratory application because of the cumbersome sample pretreatment and their qualitative not quantitative nature [7]. Chemiluminescence immunoassay (CLIA) technology is a considerable quantitative detection method, which can directly carry out dynamic quantitative detection of specific IgM and IgG antibodies. It has the advantages of high sensitivity, strong specificity, and high reliability. It is a relatively reliable detection method for TORCH infection screening [8]. Luminol has the advantages of easy synthesis, a simple structure, and good water solubility [9,10], Jiang X, Liu J, Favero et al. found that the chemiluminescence (CL) system of luminol–hydrogen peroxide is widely used in the detection of sensitive proteins, hepatitis C virus, and Manson’s schistosome eggs [11,12,13]. However, its detection limit and sensitivity are limited.

Gold nanoparticles, due to their unique photoelectric properties, can bind to a variety of biological macromolecules. Their terminals can be easily modified with various active or indicator groups (such as amino, phosphate, sulfur, fluorescent markers, electrochemical markers, etc.). They possess special properties that are incomparable to other nanomaterials [14], such as ease of immobilization or detection. As a result, gold nanoparticles are widely used in biosensors [15,16], immunoprinting technology [17], dot immunogold and silver staining techniques, homogeneous sol particle immunoassays [18], and other technologies. They provide a basis for achieving fast and accurate quantitative detection.

In this study, gold nanoparticles (Au NPs) were combined with a luminol–H_2_O_2_ chemiluminescence system by using their special optical properties and catalysis. By optimizing the experimental conditions, TORCH antibodies in a sample serum were detected by chemiluminescence immunoassay, the chemiluminescence effect of the luminol–H_2_O_2_ system enhanced with Au NPs was analyzed, and the performance of the new chemiluminescence system was evaluated. It is of great significance to improve the detection sensitivity, broaden the scope of practical application, and develop new chemiluminescence systems.

## 2. Results

### 2.1. Characterization of Au NPs

**Figure 1:**
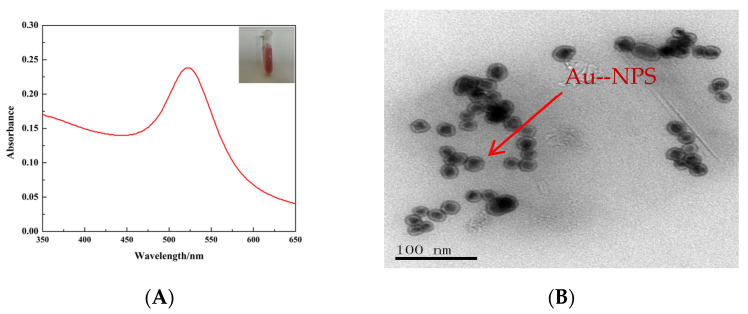
(**A**) UV–vis spectrum of Au NPs; (**B**) TEM image of Au NPs.

The prepared Au-NP solution appeared wine red in color. The UV–vis showed that the maximum absorption wavelength of the prepared Au NPs was 525 nm (Figure 1A). To obtain TEM (transmission electron microscope) images, a transmission electron microscope was selected with an accelerating voltage of 200 kV and a low current density of 5–10 pA/cm^2^. The images were captured at magnifications ranging from 50,000× to 100,000× with a scanning time of 1–2 s/frame. The obtained TEM images were then converted into grayscale images and subjected to binarization processing. Next, contour detection methods were employed to identify the boundaries of the gold nanoparticles and calculate their areas. Based on the calculated areas, the equivalent diameters of the nanoparticles were determined. Statistical analysis was performed to obtain information such as the number of nanoparticles, their average diameter, and the standard deviation. This process enables the acquisition of accurate size distribution statistics for the nanoparticles. The TEM showed that the Au NPs were uniform spherical shapes, and the sizes were approximately 10~50 nm (Figure 1B).

### 2.2. Optimization of Experimental Conditions

#### 2.2.1. Effect of the Au-NP Curing Time on the Luminescence Value of the System

**Figure 2:**
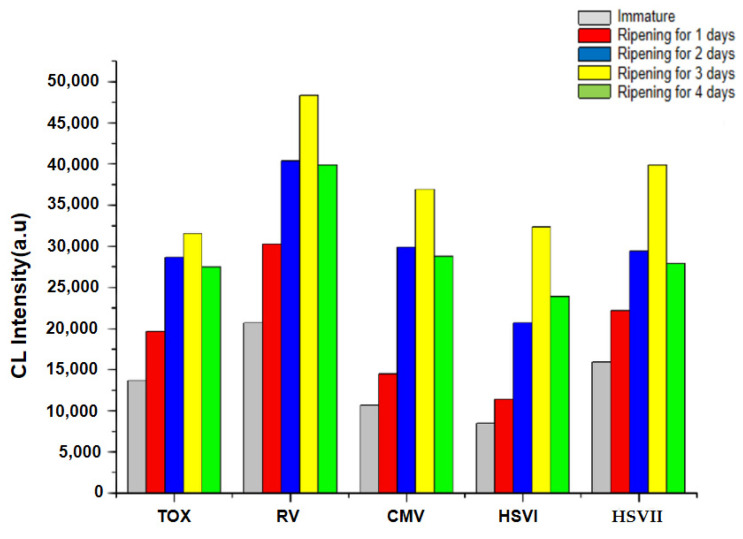
Effect of curing time on catalytic activity of Au NPs.

As a luminescence-enhanced material, the curing time of the Au NPs has great influence of the detection. The positive control sample (ORCH Positive Control Standard) was used to detect the luminescence value (chemiluminescence intensity) in strict accordance with the testing standard operating procedure (SOP). It can be seen from Figure 2 that the Au NPs that matured for 3 days have the best catalytic activity.

#### 2.2.2. The Effect of the Gold-Nanoparticle Concentration on the Luminescence Value of the System

**Figure 3:**
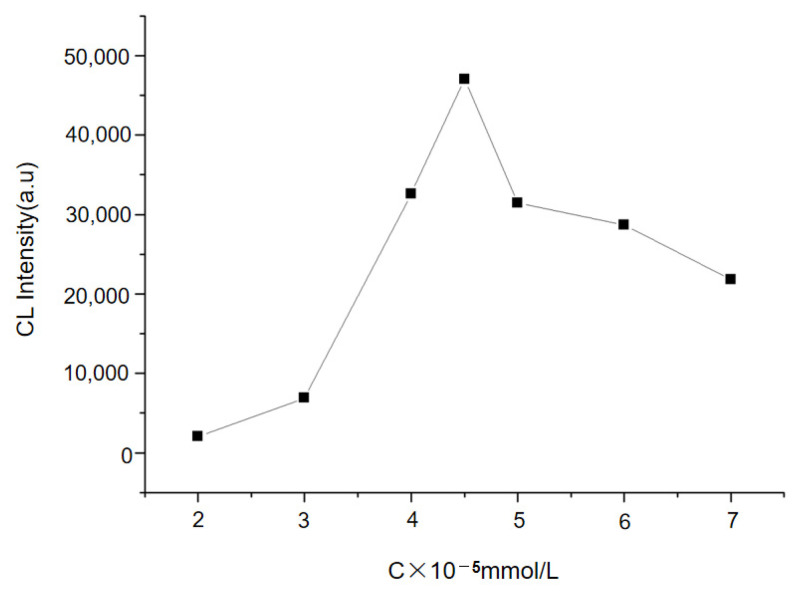
Effect of Au-NPs concentration on the luminescence value of the system.

Different concentrations (2~8 × 10^−5^ mmol/L) of Au NPs were added to the luminol–H_2_O_2_ luminescence system, and the absorbance of the positive control sample was detected. The absorbance value increased with the increase of the Au-NP concentration when the concentrations were lower than 4.5 × 10^−5^ mmol/L. However, when the concentration was higher than 4.5 × 10^−5^ mmol/L, the absorbance value decreased (Figure 3). Thus, the Au-NP concentration was optimized as 4.5 ×10^−5^ mmol/L.

#### 2.2.3. The Effect of the Addition Sequence of Gold Nanoparticles on the Luminescence Value of the System

In order to explore the influence of the addition order of the luminescence system, the well-prepared Au NPs were strictly operated according to the detection SOP, and the luminescence value was detected in TORCH experiment before, during, and after the addition of luminol. The one-way ANOVA of the order of Au-NP addition on the luminescence value of the system is shown in Table 1. According to the statistical analysis, there is no difference in the luminescence value of the system with the change of the order of Au-NP addition, so the order of Au-NP addition has no significant effect on the luminescence system. In this experiment, the Au-NP solution was added after luminol was added.

### 2.3. Enhancement of Chemiluminescence by Au NPs

We treated the positive control sample as a patient sample for inspection, compared the absorbance values of Au NPs with and without Au NPs in the luminol–H_2_O_2_ luminescent system, and statistically tested the absorbance values of Au NPs with and without Au NPs by paired *t*-test. The results are shown in Table 2. For the detection of TOX, RV, CMV, HSVI, and HSVII, there were significant differences between the methods with and without Au NPs (*p* < 0.001). In addition, the $\bar{x}$ ± s of the luminescence value with Au NPs is higher than that without Au NPs, indicating that the absorbance value of luminol–H_2_O_2_ luminescence system with Au NPs is significantly improved.

### 2.4. Establishment of the Cut-Off Value of TORCH Antibodies

In clinical practice, the detection of positive TORCH antibodies is one of the indicators for diagnosing TORCH-related viral infections. This study used the commonly used TORCH test kit in clinical settings as the standard for TORCH detection. The cut-off value is the quantity of the analyte being tested, used to determine whether the result is above or below the clinical or analytical decision point. The cut-off values for detecting TOX, RV, CMV, HSVI, and HSVII antibodies with the TORCH kit are 11,298, 7082.76, 1723.26, 4728.78, and 513.24, respectively. The cut-off values for detecting TOX, RV, CMV, HSVI, and HSVII antibodies using Au-NP/luminol–H_2_O_2_ are 14,896.98, 10,766.28, 2123.94, 8310.12, and 954.24, respectively, as shown in Table 3.

### 2.5. Actual Sample Detection

#### 2.5.1. The Reliability of the Au-NPs Luminol–H_2_O_2_ Method for TORCH Antibody Detection

A total of 127 clinical samples [Chenzhou, Hunan, 2021, Ethics Approval No. K202101401] were compared using the Au-NP-enhanced luminol luminescence method and TORCH kit to determine the reliability of the Au-NP-enhanced luminol luminescence method in the detection of TORCH antibodies. The test results of TOX, RV, CMV, HSVI, and HSVII using the two methods are shown in Table 4. The paired χ^2^ test is used to analyze the result judgment of the two analysis methods. The results showed that there was no significant difference between the Au-NP-enhanced luminol chemiluminescence and the TORCH kit in the detection of TOX antibodies (*p* > 0.05); the results of TOX antibody test items showed that the sensitivity was 84.6% and the specificity was 94.7%. The results of RV antibody test items showed that the sensitivity was 83.3% and the specificity was 96.5%. The results of the CMV antibody test items showed that the sensitivity was 90.9% and the specificity was 96.6%. The results of the HSVI antibody test items showed that the sensitivity was 85.7% and the specificity was 97.3%. The results of the HSVII antibody test showed that the sensitivity was 94.6% and the specificity was 95.6%.

#### 2.5.2. Comparison Between Non Au-NPs/Luminol–H_2_O_2_ and TORCH Kit

A total of 127 clinical samples were compared with TORCH antibody detection using the non-Au-NP/luminol–H_2_O_2_ method and TORCH kit. From Table 5, the sensitivity and specificity of the Non-Au-NPs/luminol–H_2_O_2_ method in the detection of TOX, RV, CMV, HSVI, and HSVII were obtained. A paired *x*^2^ test was used to analyze the result judgment of the two analysis methods. The results showed that the non-Au-NP/luminol–H_2_O_2_ and TORCH kit had significant differences in the detection of TORCH antibodies (*p* < 0.05); the results of TOX antibody test items showed that the sensitivity was 69.2% and the specificity was 87.7%. The results of RV antibody test items showed that the sensitivity was 66.7% and the specificity was 88.7%. The results of CMV antibody test items showed that the sensitivity was 72.7% and the specificity was 89.7%. The results of HSVI antibody test items showed that the sensitivity was 64.3% and the specificity was 86.7%. The results of the HSVII antibody test showed that the sensitivity was 61.5% and the specificity was 86.8%.

### 2.6. Performance of Au-NP-Enhanced Luminol Chemiluminescence Method

**Figure 4:**
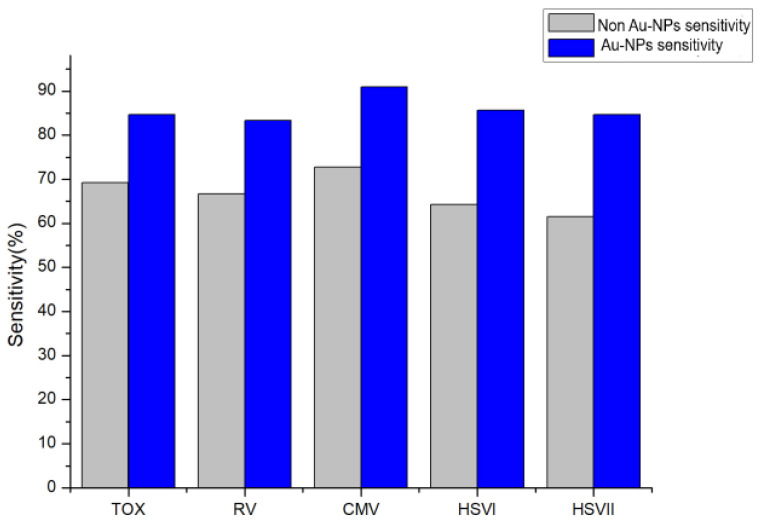
Comparison of sensitivity between Au-NP/luminol–H_2_O_2_ and non Au-NP/luminol–H_2_O_2_.

**Figure 5:**
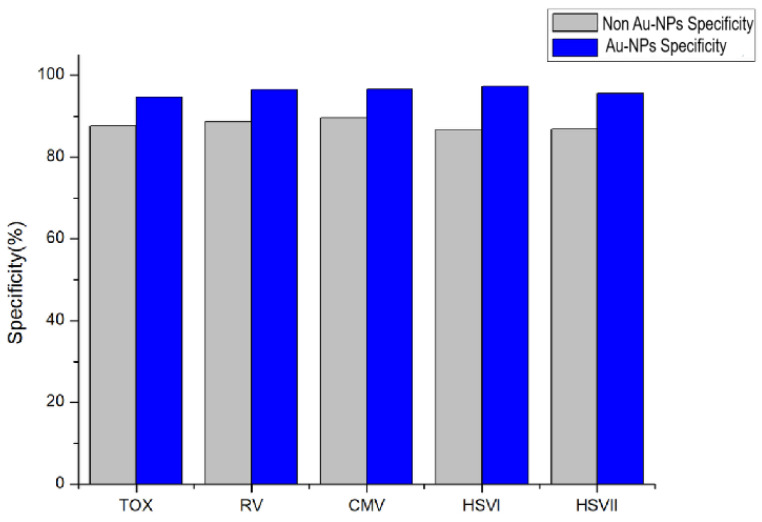
Comparison of specificity between Au-NP/luminol–H_2_O_2_ and non Au-NP/luminol–H_2_O_2_.

As shown in Figure 4 and Figure 5, the Au-NP/luminol–H_2_O_2_ has higher sensitivity and specificity than non-Au-NP/luminol–H_2_O_2_ in the detection of TOX, RV, CMV, HSVI, and HSVII antibodies.

## 3. Discussion

### 3.1. Characterization of Gold Nanoparticles

According to the small size effect of nanoparticles, the UV–vis absorption peak position, half width height and peak intensity of nanoparticles are significantly related to the concentration, volume and size distribution of nanoparticles. The size of nanoparticles can be roughly determined by the position of absorption peak. When the position of absorption peak shifts blue, the particle size decreases; On the contrary, if the position of the absorption peak redshifts, it increases accordingly. The particle size distribution becomes more and more extensive with the increase of the half width and height of the absorption peak. If the position of the absorption peak is constant, only the intensity of the absorption peak changes, indicating that the content of nanoparticles in the solution increases and the content of nanoparticles in the unit volume solution increases. The maximum absorption wavelength of nano gold prepared in the experiment is 525 nm, the absorption peak is single peak, and the peak shape is narrow and symmetrical; The color of colloidal gold solution depends on the distance between particles to a great extent. When the distance between particles is greater than the average particle diameter, the solution turns red; When the distance between particles is less than the average particle diameter, the solution is blue. Therefore, it can be suggested that the Au NPs prepared by us have good dispersion and no agglomeration [19,20].

The transmission electron microscope of gold nano solution shows that the nanoparticles prepared in the experiment are spherical, the diameter is about 10~50 nm, there is no obvious agglomeration, and the dispersion is good. The reason may be that the interaction force between particles in the solution is weak. Even if the size of nano gold particles prepared by us is small, it will not agglomerate.

### 3.2. Optimization of Experimental Conditions

Particle ripening, also known as Ostwald ripening, refers to that when large and small particles exist together in the solution, due to the higher chemical potential of small particles, that is, small nanoparticles have greater solubility, when the monomer concentration in the solution is low, small particles will dissolve, large particles will continue to grow, and the polydispersity of particles will increase [21,22]. The experimental results show that the luminescence values of TOX, RV, CMV, HSVI and HSVII reach the maximum after 3 days of aging of nano gold. Then, with the extension of aging, the luminescence value of the system gradually decreases. Therefore, it is determined that nano gold after 3 days of aging has the best catalytic activity. The reason may be that during the synthesis of gold nanoparticles, as the monomer concentration decreases to a certain extent, some atoms in gold nanoparticles migrate from one part of the particles to another, and this migration process takes a certain time, that is, aging [23,24]. In this study, after 3 days of aging, the gold nanoparticles have been fully formed, and the luminescence value of luminol–H_2_O_2_ system is the highest. However, when the aging time is too long, the luminescence value of luminol–H_2_O_2_ system is reduced due to its unstable characteristics.

The concentration of gold nanoparticles significantly affects the chemiluminescence enhancement performance of the system. In this study, the optimal concentration level of gold nanoparticles is 4.5 × 10^−5^ mmol/L. When its concentration is between 2~10 × 10^−5^ mmol/L When the concentration is between 10^−5^ mmol/L, the change of concentration level can make the absorbance value fluctuate. When the concentration of gold nanoparticles ≤4.5 × 10^−5^ mmol/L, the absorbance value is positively correlated with the concentration of gold nanoparticles; If >4.5 × 10^−5^ mmol/L, due to the interaction between gold nanoparticles and silver particles, the energy generated by chemiluminescence moves back and forth between the particles, resulting in the decrease of absorbance value. With the increasing concentration of gold nanoparticles, the absorbance value will not fluctuate significantly.

With the change of the addition order of gold nanoparticles, the luminescence value of the system did not change significantly. According to the chemiluminescence reaction mechanism of luminol–H_2_O_2_ catalyzed by nanoparticles, nanoparticles only act as the nucleation center in luminol–H_2_O_2_ system, which can catalyze luminol to reduce metal cations to metal atoms. At the same time, luminol is oxidized to luminol radical, and the generated luminol radical further reacts with reactive oxygen species to produce chemiluminescence. Therefore, the addition order of gold nanoparticles has no effect on the luminescence value of the system. Based on the operator’s convenient choice of experimental operation, gold nanoparticles were added after luminol.

### 3.3. Mechanism of the Enhanced Chemiluminescence of Gold Nanoparticles

In luminol–H_2_O_2_ system, H_2_O_2_ is catalyzed by gold nanoparticles, and the O-O bond of hydrogen peroxide has the possibility of breaking, resulting in two HO-free radicals on the surface of gold nanoparticles. At the same time, some electrons move between this product and gold nanoparticles and show a relatively stable state [25,26]. Based on this, HO^−^ radicals continue to combine hydrogen peroxide anion and luminol anion in alkaline solution to form O_2_^−^ (superoxide anion radical) and L-(luminol radical), which greatly improves the formation rate of these two kinds of radical products. Under the catalysis of gold nanoparticles, electron transfer also occurs between O_2_^−^ and L-formed on the surface of gold nanoparticles, resulting in important peroxide intermediates, which are decomposed to obtain the final product of 3-aminophthalate ion, thus forming chemiluminescence [27,28]. Through the above analysis, it can be seen that Au NPs enhance the chemiluminescence signal of the system by participating in the formation of free radicals in the system and catalyzing the electron transfer of free radicals.

In this study, the paired *t*-test was used to statistically test the absorbance values of gold nanoparticles and non gold nanoparticles. The results show that there is a statistical difference between the two methods of adding gold nanoparticles and non gold nanoparticles in the detection of TOX, RV, CMV, HSVI and HSVII *(p* < 0.001). It can be considered that there is a difference between the luminol–H_2_O_2_ luminescence value after adding gold nanoparticles and that without gold nanoparticles. It can be seen from $\bar{x}$ ± s that the luminescence value of gold nanoparticles is significantly higher than that of non gold nanoparticles. At the same time, the CV value shows that the luminol chemiluminescence system of gold nanoparticles has good stability.

### 3.4. Actual Sample Detection

The TORCH antibody was detected by nano-gold-enhanced luminol luminescence method and TORCH kit commonly used in clinic. The results of the two methods were analyzed by paired *x*^2^ test. The results showed that there was no significant difference between Au-NP-enhanced luminol chemiluminescence and TORCH kit in the detection of TOX antibody (*p* = 0.289); There was no significant difference between Au-NP-enhanced luminol chemiluminescence and TORCH kit in the detection of RV antibody (*p* = 0.688); There was no significant difference between Au-NP-enhanced luminol chemiluminescence and TORCH kit in the detection of CMV antibody (*p* = 0.375); There was no significant difference between Au-NP-enhanced luminol chemiluminescence and TORCH kit in the detection of HSVI antibody (*p* = 0.999); There was no significant difference between Au-NP-enhanced luminol chemiluminescence and TORCH kit in the detection of HSVII antibody (*p* = 0.453); Therefore, it can be considered that the nano-gold-enhanced luminol luminescence method for the detection of TORCH antibody is accurate and reliable.

The sensitivity and specificity of Au-NP-enhanced luminol chemiluminescence method are significantly higher than those without Au-NP enhancer. The main reason is that Au-NPs are added to luminol–H_2_O_2_ system, participate in the formation of HO- free radicals in luminol–H_2_O_2_ system, catalyze the transfer of free radical electrons, and greatly enhance the chemiluminescence signal of the whole system. Therefore, the new chemiluminescence system can better provide help for TORCH detection.

### 3.5. Research Prospects

Nanogold possesses unique electrochemical advantages, but it also has some limitations and drawbacks. Compared to traditional methods, nanogold research may involve higher analytical costs. Toxicity, biocompatibility, and environmental sustainability require further investigation. In the future, consideration can be given to extracting gold from waste solutions in routine practice, further promoting the development of nanogold research, expanding its application fields, and fostering its widespread use in areas such as medicine, biosensing, and environmental protection.

## 4. Materials and Methods

### 4.1. Reagents and Instruments

127 serum samples (Serum was obtained by centrifuging blood samples at 3000 rpm for 3 min). Hydrogen peroxide (H_2_O_2_, 30%, Aladdin, Shanghai, China), luminol (98%, Aladdin, Shanghai, China), Tetrachloroauric acid (HAuCl_4_, 99.9%, Aladdin, Shanghai, China). Ultraviolet visible spectrophotometer (UV-2450, Shimadzu, Suzhou, China), The scanning electron microscope (SEM, TESCAN MIRA LMS, Tescan, Brno, Czech Republic). transmission electron microscope (TEM, JEM3010, Japanese electronics, Tokyo, Japan) Luminescent immunoanalyzer (JETLIA-962, Beijing Yuande, Beijing, China), microplate reader (ADC-elisa-400, Beijing Yuande, Beijing, China).

### 4.2. Preparation of Au NPs

Add 1.65 mL of 2% chloroauric acid into a conical flask, add an appropriate amount of water, place the conical flask in an oil bath to avoid light reflux, and stir with magnetic force at the same time. When the solution boils, add 12.8 mL of 38.8 × 10^−3^ mol/L trisodium citrate solution immediately, and maintain boiling for 15 min. Remove the heat source, stir until cool, and obtain the wine-red dispersed Au-NP solution. After the Au-NP solution is filtered by 0.22 μm needle-type water-soluble filter, the prepared gold nano solution is obtained [10]. Characterization of Au NPs is obtained using UV–vis and an transmission electron microscope (TEM). The maximum absorption wavelength of the prepared Au-NP solution was determined. The morphology and particle sizes of the Au NPs were observed.

### 4.3. Evaluation of the Enhanecment of Au NPs on Luminol–H_2_O_2_ Luminescence System

Au NPs (2~8 × 10^−5^ mmol/L) were studied according to the different curing times (0, 1, 2, 3, and 4 days) before, during, and after the addition of luminol. The sensitivity and specificity were analyzed to establish the cut off value of Au-NP-enhanced luminol chemiluminescence for the detection of TORCH antibodies.

### 4.4. Detection of TORCH

**Figure 6:**
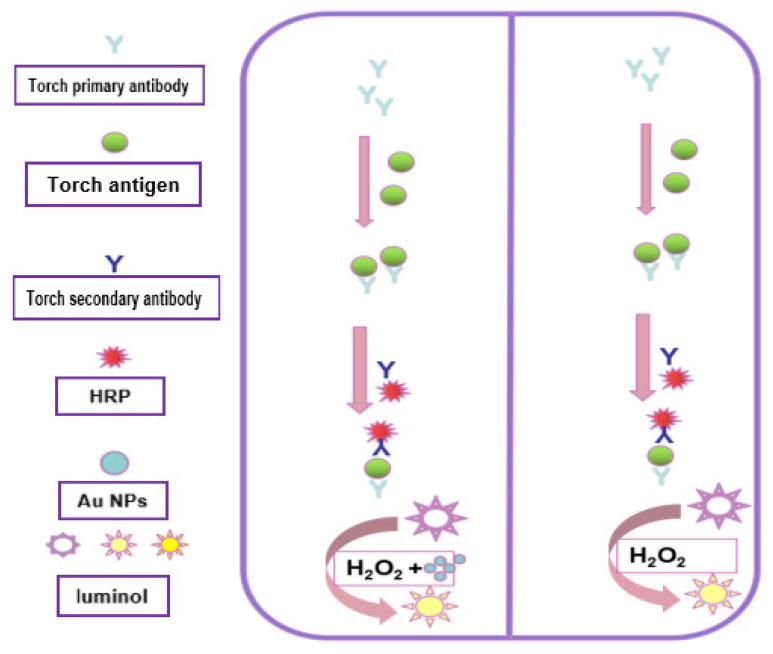
Process of Au-NP-enhanced luminol chemiluminescence detection of TORCH antibodies.

**Figure 7:**
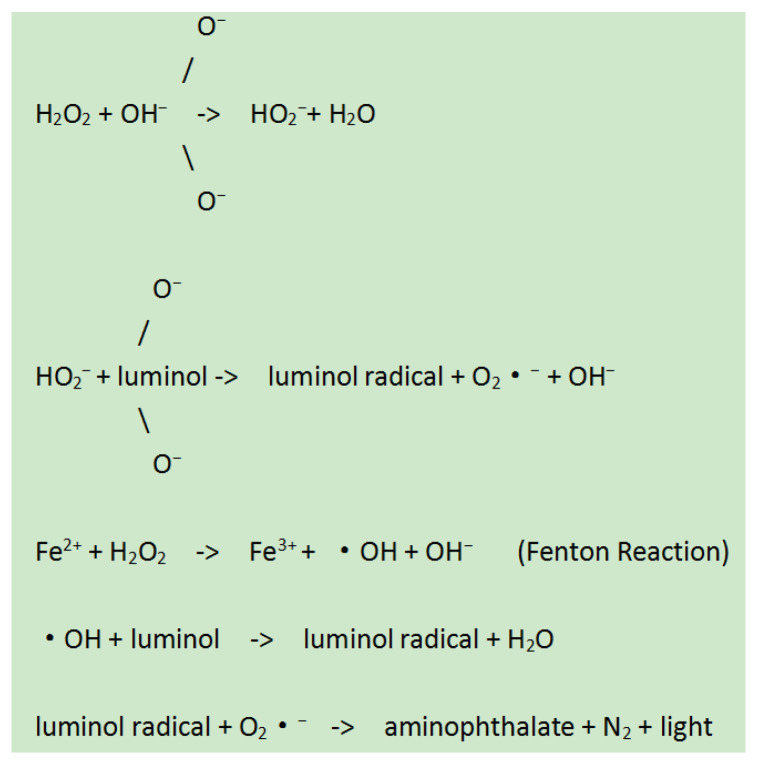
Diagram for formation of HO- free radicals in luminol–H_2_O_2_ system.

For the detection of TORCH, first, Add 50 μL positive and negative controls to their respective wells, and add 50 μL diluted sample to each corresponding sample well. Then, oscillate for 5 s or more and place in a water bath (37 °C) for 30 min for incubation treatment. Pour out or suction the reaction solution, and after standing for at least 30 s, add washing solution (0.3 mL) for 5 cycles of washing, tap the plate, and add 50 μL of the reaction antigen and 50 μL of the enzyme (Horseradish peroxidase, HRP) conjugate to each hole in turn. After incubating and washing the plate, add the luminol solution, Au-NP solution, and hydrogen peroxide solution, 50 μL each (100 μL in total), vibrate for no less than 5 s, and place it in the dark for 5 ± 0.5 min. Use the MPC-1 microporous plate single photon counter to measure the luminescence intensity of positive control, negative control, and test samples. The process diagram for detecting TORCH antibodies using gold-nanoparticle-enhanced luminol chemiluminescence is shown in Figure 6. Diagram for formation of HO- free radicals in luminol–H_2_O_2_ system is shown in Figure 7.

### 4.5. Data Analysis

SPSS 19.0 analysis software was used for statistical processing, *t*-test of paired design and one-way ANOVA were used for measurement data, and χ^2^ test was used for paired comparison of enumeration data, where *p* ≤ 0.05 was statistically significant.

## 5. Conclusions

The test results of actual clinical samples show that there is no statistically significant difference between this method and the test results of the TORCH kit commonly used in clinics. It can be considered that the Au-NP-enhanced luminol luminescence method is accurate and reliable for the detection of TORCH antibodies and can be used for the determination of clinical eugenics antibodies.

The catalytic activity of gold nanoparticles combined with the classical luminol hydrogen peroxide chemiluminescence system was used for the analysis and determination of TORCH, and the experimental conditions were optimized. The results show that the aging time of gold nano is 3 days and the concentration of gold nano of 4.5 × 10^−5^ mmol/L was the best experimental condition. The results of the TOX antibody test showed that the sensitivity was 84.6% and the specificity was 94.7%. The results of the RV antibody test showed that the sensitivity was 83.3% and the specificity was 96.5%. The results of the CMV antibody test showed that the sensitivity was 90.9% and the specificity was 96.6%. The results of the HSVI antibody test showed that the sensitivity was 85.7% and the specificity was 97.3%. The results of the HSVII antibody test showed that its sensitivity was 84.6% and specificity was 95.6%.

From the aspects of absorbance, sensitivity, and specificity, it shows that gold nanoparticles can well enhance luminol–H_2_O_2_ luminescence system. The luminol chemiluminescence system enhanced by gold nanoparticles will be well used in the detection of eugenics.

## Figures and Tables

**Table 1:** One way ANOVA of the effect of the Au-NP addition order on the luminescence value of the system.

Target	Pre-RLU	Mid-RLU	Post-RLU	*F*	*p*
TOX	38,550 ± 380.99	38,516 ± 407.87	38,478 ± 467.74	0.037	*p* = 0.964
RV	35,716 ± 330.23	36,035 ± 633.60	35,875 ± 134.16	0.722	*p* = 0.506
CMV	27,746 ± 356.00	27,483 ± 477.50	27,892 ± 194.91	1.634	*p* = 0.236
HSVI	28,522 ± 419.71	28,707 ± 536.70	28,232 ± 413.10	1.385	*p* = 0.288
HSVII	38,565 ± 332.08	38,645 ± 430.83	38,110 ± 443.00	2.538	*p* = 0.120

Pre-RLU: Add luminol first, followed by Au NPs, to detect luminescence values. Mid-RLU: Au NPs and luminol are simultaneously added to detect luminescence values. Post-RLU: Add Au NPs first, then add luminol to detect the luminescence value.

**Table 2:** Paired *t*-test of absorbance values of Au NPs and non-Au NPs.

Target	Groups	*n*	$\bar{x}$ ± s	*t*	*P*	cv%
TOX	Non-Au NPs	20	30,904.90 ± 1173.86	31.076	<0.001	3.80 3.70
	Au NPs	20	42,664.60 ± 1578.67			
RV	Non-Au NPs	20	23,302.60 ± 1621.87	29.409	<0.001	6.96 5.50
	Au-NPs	20	34,697.10 ± 1908.67			
CMV	Non-Au NPs	20	15,873.35 ± 616.36	63.453	<0.001	3.86 3.07
	Au-NPs	20	26,791.05 ± 823.93			
HSVI	Non-Au NPs	20	17,661.30 ± 578.22	46.351	<0.001	3.27 3.60
	Au-NPs	20	27,876.70 ± 1003.70			
HSVII	Non-Au NPs	20	26,501.15 ± 884.03	38.285	<0.001	3.34 3.30
	Au-NPs	20	36,975.15 ± 1220.92			

**Table 3:** Cut off value detected by TORCH kit and Au NP/luminol–H_2_O_2_.

Target	RLU Absorbance		$\mathrm{RLU}\;(\bar{x}$ ± s)		Cut-Off	
	TORCH Kit	Au-NPs/Luminol–H_2_O_2_	TORCH Kit	Au-NPs/Luminol–H_2_O_2_	TORCH Kit	Au-NPs/Luminol–H_2_O_2_
TOX	5375	7125	5380.00 ± 131.91	7093.80 ± 57.01	11,298.00	14,896.98
	5305	7102				
	5518	7035				
	5204	7039				
	5498	7168				
RV	3725	5158	3715.60 ± 117.40	5126.80 ± 112.32	7802.76	10,766.28
	3867	4968				
	3549	5166				
	3765	5268				
	3672	5074				
CMV	808	989	820.60 ± 10.61	1011.40 ± 37.71	1723.26	2123.94
	821	1076				
	814	1011				
	836	982				
	824	999				
HSVI	2323	4088	2251.80 ± 78.32	3957.20 ± 151.62	4728.78	8310.12
	2142	3698				
	2296	4038				
	2197	3980				
	2301	3982				
HSVII	241	498	244.40 ± 8.51	454.40 ± 38.91	513.24	954.24
	245	421				
	232	456				
	254	410				
	250	487				

RLU (relative light unit).

**Table 4:** Detection of TORCH antibody by Au-NPs luminol–H_2_O_2_ and TORCH Kit.

Target	Au-NPs Luminol–H_2_O_2_	TORCH Kit		Subtotal	Evaluate
		+	−		
TOX	+	11	6	17	Sensitivity = 84.6% Specificity = 94.7%
	−	2	108	110	
	total	13	114	127	
	*p* value	*p* = 0.289			
RV	+	10	4	14	Sensitivity = 83.3% Specificity = 96.5%
	−	2	111	113	
	total	12	115	127	
	*p* value	*p* = 0.688			
CMV	+	10	4	14	Sensitivity = 90.9% Specificity = 96.6%
	−	1	112	113	
	total	11	116	127	
	*p* value	*p* = 0.375			
HSVI	+	12	3	15	Sensitivity = 85.7% Specificity = 97.3%
	−	2	110	112	
	total	14	113	127	
	*p* value	*p* = 0.999			
HSVII	+	11	5	16	Sensitivity = 84.6% Specificity = 95.6%
	−	2	109	111	
	total	13	114	127	
	*p* value	*p* = 0.453			

**Table 5:** Detection of TORCH antibodies by non-Au-NPs luminol–H_2_O_2_ and TORCH Kit.

Target	Non Au-NPs Luminol–H_2_O_2_	TORCH Kit		Subtotal	Evaluate
		+	−		
TOX	+	9	14	23	Sensitivity = 69.2% Specificity = 87.7%
	−	4	100	104	
	total	13	114	127	
	*p* value	*p* = 0.031			
RV	+	8	13	21	Sensitivity = 66.7% Specificity = 88.7%
	−	4	102	106	
	total	12	115	127	
	*p* value	*p* = 0.049			
CMV	+	8	12	20	Sensitivity = 72.7% Specificity = 89.7%
	−	3	104	107	
	total	11	116	127	
	*p* value	*p* = 0.035			
HSVI	+	9	15	24	Sensitivity = 64.3% Specificity = 86.7%
	−	5	98	103	
	total	14	113	127	
	*p* value	*p* = 0.041			
HSVII	+	8	15	23	Sensitivity = 61.5% Specificity = 86.8%
	−	5	99	104	
	total	13	114	127	
	*p* value	*p* = 0.041			

## Data Availability

Data are contained within the article.
